# On Vibronic-Driven Action and Mechanosensitive G Protein-Coupled Receptors

**DOI:** 10.3390/ijms27052262

**Published:** 2026-02-27

**Authors:** Zong Jie Cui, Wei Mei Huang, Peng Juan Li, Xiao Bing Xie

**Affiliations:** 1 Department of Biology, College of Life Sciences, Beijing Normal University, Beijing 100875, China; 2 Ministry of Education Laboratory for Cell Proliferation and Regulation Biology, College of Life Sciences, Beijing Normal University, Beijing 100875, China

**Keywords:** molecular jackhammers, G protein-coupled receptors (GPCR), calcium signaling, photovibronic-driven action (PVA), cholecystokinin 1 receptors (CCK1R), neuropeptide Y2 receptor

## Abstract

G protein-coupled receptors (GPCR) are targeted by more than one third of the clinically available drugs as specific ligands. Important as GPCR ligands may be, cases of ligand-independent GPCR activation are also abundant. In a recent article published in the journal *ACS Nano*, a series of cyanine skeleton-based plasmonic molecules, or molecular jackhammer, as the authors christened them, after insertion into the plasma membrane, was found after excitation by far-red light (730 nm) to vibronically-driven activate Gq-mediated calcium signaling in four different cell lines of both epithelial and muscle cell origin, which is normally activated after agonist stimulation of Gq-coupled GPCR, but the possible involvement of GPCR remains to be examined. This novel mode of activation of calcium signaling, normally associated with agonist-stimulated GPCR activation, is compared to nanoclustering activation of the neuropeptide Y2 receptor and photodynamic activation of the cholecystokinin 1 and 2 receptors.

## 1. Introduction

Cellular physiological functions are executed by dedicated functional proteins. According to the International Union of Pharmacology, these functional proteins could be divided into at least six major categories, including G protein-coupled receptors (GPCR), ionic channels, transmembrane transporters, and others, with GPCR being among the largest families of functional proteins [1]. Specific GPCR ligands account for a predominant proportion of all clinically available drugs; the large arsenal of small molecule ligands for GPCR have therefore been extensively studied for over a century, providing expanding repertoires to counter human and animal diseases [2]. In view of the importance of GPCR in the normal function of different regulatory systems of the human life process, it is unsurprising that ligand-independent activation and modulation of different sub-families of GPCR has drawn considerable attention in recent years [3,4]. GPCR function has been found to be regulated by temperature [5], by the lipid microenvironment of the plasma membrane (such as cholesterol content) [6], by trans-membrane potential [7], by forced nanoclustering of receptor proteins [8,9], by mechanical force exerted via magnetic field-driven magnetic nanodiscs [10], and by localized concentration of the excited delta state molecular singlet oxygen (Δ^1^O_2_) [3,11].

## 2. Photovibronic-Driven Activation of Gq-Coupled Calcium Signaling

In a recent article published in the journal *ACS Nano*, it was found that a class of light-responsive molecules, the so-called plasmonic molecular jackhammers, when applied to either the widely used epithelial cell line HEK293, or to muscle cell lines of the skeletal, cardiac, or smooth muscle cell origin, could trigger immediate increases in cytosolic calcium concentration after a brief pulse (0.2–0.8 s) of far-red (730 nm) laser light irradiation (a diode laser at an output power of 0.42 mW in a light spot with a diameter of 5 μm, with a calculated power density of 2140 Watt/cm^2^) [12].

**Figure 1:**
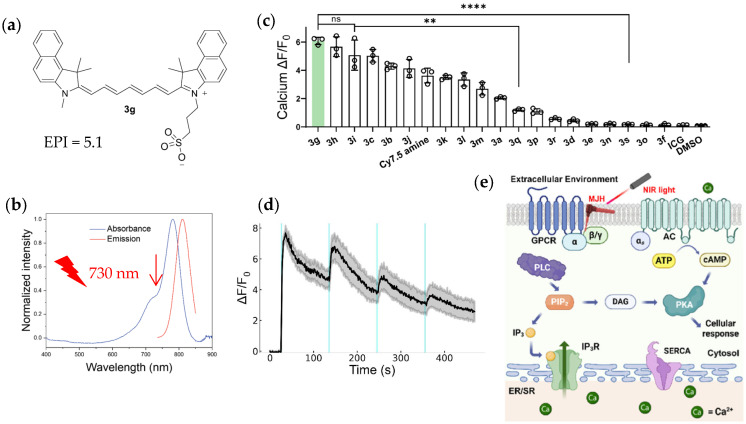
Activation of calcium signaling by far-red light-driven molecular jackhammers, which is normally associated with agonist-stimulated GPCR activation. (**a**) Structure of N-methyl, N’-sulphonate-benzoindolenine Cy7.5 or 3g, with an experimental plasmonicity index (EPI) of 5.1. (**b**) The absorption and emission spectra of 3g in methanol. Note the vibronic shoulder in the absorption spectrum indicated by the downward red arrow; this is where the excitation light (at 730 nm) was applied to 3g. (**c**) Efficacy of a set of molecular jackhammers to photovibronic-drivenly increase the cytosolic calcium concentration in HEK293 cells, as measured by Fluo-4 confocal imaging, compared to reference compound 3g (indicated by a light green background). The double (**) or quadruple (****) asterisks indicate statistical significance. The abbreviation of “ns” indicates statistically not significant. (**d**) Far-red light-driven increase in cytosolic calcium in 3g-incubated HEK293 cells could be repeated multiple times (4 times are shown, the light pulses as indicated by vertical light green lines). (**e**) Vibronic-driven activation of calcium signaling (that is otherwise normally associated with activation of Gq-coupled GPCR) in 3g-incubated HEK293 cells. Figure panels (**a**–**e**) are adapted from [12] with permission from The American Chemical Society. Abbreviations used in this figure are listed alphabetically, as follows: AC, adenylate cyclase; DAG, diacylglycerol; DMSO, dimethyl sulfoxide; EPI, experimental plasmonic index; ER/SR, endoplasmic/sarcoplasmic reticulum; GPCR, G protein-coupled receptors; ICG, indocyanine green; IP_3_, inositol-1,4,5-trisphosphate; IP_3_R, inositol-1,4,5-trisphosphate; MJH, molecular jackhammers; and SERCA, sarcoplasmic/endoplasmic reticulum Ca^2+^-ATPase.

It was found that after a brief pulse of laser irradiation, these molecular jackhammers induced immediate increases in cytosolic calcium concentration, reaching a calcium peak value within 13 s in HEK293 cells, but structurally similar non-plasmonic negative control molecules (non-polar poly-ene or a di-cationic state molecule) had no such effect on cytosolic calcium. A series of 19 newly synthesized benzoindolenine-substituted Cy7.5 core molecules were examined, together with a previously commercially available plasmonic molecule of Cy7.5 amine and the negative control indocyanine green (ICG). One compound, N-methyl, N′-sulphonate-benzoindolenine Cy7.5 or rather 3g (Figure 1a,b), as the authors named for brevity, with one of the highest experimental plasmonicity indexes (EPI = 5.1) [12,13], was found to be particularly efficient at inducing cytosolic calcium increases (Figure 1c). The calcium-enhancing effects of such plasmonic molecules were found not due to the generation of reactive oxygen species—antioxidants being without any effect on jackhammer-induced calcium increases. Such plasmonic molecular jackhammers did not exert any detectable increase in temperature in the microenvironment after far-red light illumination [12].

These workers examined the subcellular distribution of the molecular jackhammer 3g—notable co-localization with the plasma membrane was found. The calcium-increasing effect after far-red light illumination of 3g-incubated HEK293 cells could be repeatedly produced (Figure 1d); the cellular mechanisms involved were then examined in great detail (Figure 1e). It was found that pretreatment with endoplasmic reticulum calcium-ATPase inhibitor thapsigargin, phospholipase C inhibitor U73122 or with inositol-1,4,5-trisphosphate receptor inhibitor xestospongine C all almost completely blocked the calcium increase induced by light-irradiated 3g; both Gαq inhibitor FR900359 and Gβγ inhibitor Gallein showed sizable inhibition of the calcium increase induced by light-irradiated compound 3g; protein kinase A blocker cAMPS-Rp and adenylate cyclase inhibitor also showed small but statistically significant inhibition. From these data, it was concluded that the Gq-PLC-IP_3_R-Ca^2+^ signaling pathway was activated that is normally associated with agonist-stimulated activation of Gq-coupled GPCR, but also with a minor contribution from the Gs-AC-cAMP-PKA pathway [12] (Figure 1e).

Other than the widely used epithelial cell line HEK293, the effect of far-red light illuminated 3g was also examined in three muscle cell lines—C2C12, H9c2, and A7r5—of the skeletal, cardiac, and smooth musclecell origin, respectively. Similar increases in cytosolic calcium concentration were all seen in these three different muscle cell lines, together with correlated muscle cell contractions [12]. These authors made an effort to examine the light-driven jackhammer mechanical oscillatory effect on three-dimensional organoids (diameter 200–300 μm) generated from the skeletal muscle cell line of C2C12. The effect of light-illuminated 3g was found to increase cytosolic calcium concentration at the peripheral of the organoid, which was relevant to the diffusion depth at the time of examination of 3g from the extracellular solution [12]. The effect of far-red light-irradiated 3g was compared with a blue-light (405 nm)-driven molecular–motor molecule of MM1, and the far-red light-driven molecular jackhammer 3g was found to be more suitable for mechanical manipulations of the cytosolic calcium concentration in the skeletal muscle cells of the organoids. Judging from the data with the skeletal muscle organoids, such jackhammers would probably also be more suitable for whole tissue, organ or *in vivo* situations, considering that all biological tissues have an optical window of light penetration in the far-red to infrared regions of 600–1400 nm [12,14].

## 3. Mechanosensitive G Protein-Coupled Receptors

Although these authors did not investigate the direct effect of their far-red light-driven molecular jackhammers, by high frequency stretching—contraction cycles at the resided plasma membranel, on the cell surface GPCR in the epithelial cell line HEK293 or in the muscle cell lines C2C12, H9c2, and A7r5, their data could suggest that GPCR might be activated in these different cell lines, to trigger rather similar downstream calcium signaling (Figure 1e). It is known that HEK293 express endogenous M1 acetylcholine receptors at low-density levels, as well as P2Y1and P2Y2 purinergic receptors [15] (Table 1). The muscle cell lines express their own set of GPCR. The skeletal muscle cells C2C12 express β-adrenoceptors, P2Y6 purinergic receptors, and H3 histamine receptors; the cardiomyocytes H9c2 express A1 adenosine receptors, β2-adrenoceptors, endothelin A receptors, and V1 vasopressin receptors; whereas the smooth muscle cells A7r5 express V1 vasopressin receptors and 5-HT2 receptors [16] (Table 1). Possibly to avoid any artifacts in calcium measurements associated with inherent contractions after repeated increases in cytosolic calcium concentration, all calcium measurements in this work and elsewhere by these workers were limited to a few minutes (mostly to a maximum of 2.5 min), and no inherent or physiological calcium oscillations or multiple calcium spikes were revealed after light irradiation by a single far-red laser pulse. Induction of the physiologically relevant calcium oscillations would be correlated to physiological activation of GPCR [3,11]. Such works may be done in other types of cells (such as the pancreatic acinar cells, pancreatic islet β cells, hepatocytes or others), tissues or organs showing intrinsic calcium oscillations (after activation of endogenous G protein-coupled receptors) in the future.

The basis for mechanical vibronic-driven activation of calcium signaling, normally associated with agonist-stimulated activation of GPCR, by high-frequency plasmonic oscillations at the plasma membrane, as determined by the experimental plasmonicity index (EPI, with the slope of the linear correlation between absorbance and dielectric constant multiplied by the number of −1000) of the jackhammer molecules, remains to be determined. It may be noted, however, that some of these GPCR could be mechanosensitive and be activated by the molecular jackhammers after red-light irradiation, especially GPCR in different muscle cell lines. For comparison and for clues to future research, a short list of mechanosensitive GPCR is presented in Table 1 [15,16,17,18,19].

It may be noted here that biophysical activation of different GPCR have been reported. Light-driven physical clustering (concentrating) of GPCR to a nano-domain has been reported to result in the activation of the calcium-mobilizing neuropeptide Y2 receptor (a class A GPCR), to increase cell motility and migration [8]. The ligand-independent nanoclustering-induced receptor activation was readily blocked by Y2R antagonist [8]. Interestingly, the nanoclustering-induced Y2R activation was different from neuropeptide Y agonist-stimulated receptor activation, in that only the agonist-activated modality could recruit downstream β-arrestin signaling but clustering-activated modality could not [9]. In this context, it is notable that photodynamic activation of the cholecystokinin type 1 receptor (CCK1R) by singlet oxygen (^1^O_2_) is associated with repetitive calcium spikes; the photodynamically-activated CCK1R induced calcium oscillations and associated amylase secretion, which were very similar to those induced by a physiological concentration of the endogenous agonist CCK [3,11]. Photodynamic activation of CCK1R was readily abolished by the CCK1R antagonist FK480 [3,11]. If vibronic-driven action could also directly activate the mechanosensitive GPCR, this could be compared to these two cases of biophysical GPCR activation (receptor nanoclustering activation, and photodynamic welding of the activated state/conformation). All three modalities of GPCR activation may have one thing in common—the increased probability for collision between receptors and their cognate Gq proteins to trigger downstream calcium signaling. Currently, nanoclustering activation is limited to the neuropeptide Y2 receptor [8,9] and photodynamic activation is limited to CCK1R/CCK2R but the number of GPCR activated by photodynamic action is expanding [3,11]. At present, verified GPCR activation by plasmonic molecular jackhammers, if any, remains to be identified.

## 4. Conclusions and Perspectives

A common problem with subcellular localization of small bioactive molecules is that the associated subcellular specificity is rather limited [20], as is the case of plasma membrane localization of 3g [12]. The plasma membrane localization might be further improved by increasing the extent of sulphonation of molecules such as 3g (to increase their hydrophilicity), by limiting the time period of incubation (currently at 2 μM for 45 min) with the target cells such as the case of sulphonated aluminum phthalocyanines [3,11,21], or by other means such as covalent cross-linking to target GPCR proteins. This covalent cross-linking can be achieved if the plasmonic jackhammer molecules are conjugated to the ligand molecules of certain protein tags such as HaloTag [22]. Then, protein-tag ligand-conjugated molecular jackhammers will be covalently linked to a targeted GPCR (an example would be the mechanically sensitive angiotensin II receptor type 1, AT1R) which would be expressed as a construct of HaloTag-GPCR (HaloTag-AT1R, for example), to verify whether vibronic-driven action could directly activate any GPCR. As mentioned above, nanotechnology and nano-engineering have ensured that only the neuropeptide Y2 receptors are clustered for activation [8,9]. Protein photosensitizer–GPCR constructs, such as miniSOG-CCK1R also have ensured highly specific permanent activation of CCK1R by photodynamic action [3].The protein tagging technology will also ensure probably specific vibronic-driven activation of targeted GPCR in the future. The photo-powered vibronic-driven activation method may be preferentially suited for GPCR members that are intrinsically sensitive to mechanical activation: note the reported order of mechanical sensitivity of H1 (histamine receptor type 1) > AT1 (angiotensin II receptor type 1 > M5 (muscarinic acetylcholine receptor type 5) > V1A (vasopressin receptor 1A) [23,24]. The mechanical sensitivity of GPCR has been found to be related to the presence or size of the horizontal helix 8 at the cytosolic C-terminal tail of the receptor [25]. It must not be ignored that activation of some mechanosensitive ionic channels such as Piezo, TRP and the subsequent calcium influx will also lead to increases in cytosolic calcium concentrations [24]. Finally, although it remains only an attractive possibility that vibronic-driven action could directly activate mechanosensitive GPCR before solid data are reported in the literature, some cytosolic proteins (alternative targets) have also been reported to possess special motifs to bind and activate G proteins directly in place of GPCR in vertebrate development [26,27].

## Figures and Tables

**Table 1:** Some GPCR expressed in HEK293, C2C12, H9c2 and A7r5 cells, compared to known mechanosensitive GPCR [15,16,17,18,19].

	GPCR
HEK293	M1, P2Y1, P2Y2
C2C12	β1, P2Y6, H3
H9c2	A1, β2, ETA, V1
A7r5	V1, 5-HT2
Mechanosensitive	AT1, B2, D5, ETA, FP1, H1, M5, PTH1, V1A, P2Y6, CysLT1

Abbreviations: 5-HT2, 5-hydroxytryptamine receptor 2; A1, adenosine receptor type 1; AT1, angiotensin II receptor type 1; B2, bradykinin receptor type 2; β1, β2, β-adrenoceptor 1, 2; CysLT1, cysteinyl leukotriene receptor 1; D5, dopamine receptor type 5; FP1, formyl peptide receptor 1; ETA, endothelin receptor A; H1, H3, histamine receptor type 1, 3; M1,5, muscarinic acetylcholine receptor type 1, 5; P2Y1, P2Y2, P2Y6, P2 purinergic receptors type 1,2,6; PTH1, parathyroid hormone receptor 1; and V1(A), vasopressin receptor type 1(A).

## Data Availability

No new data were created or analyzed in this study. Data sharing is not applicable to this article.

## Abbreviations

The following abbreviations are used in this manuscript:

5-HT2	5-Hydroxytryptamine receptor 2
A1	Adenosine receptor type 1
AC	Adenylate cyclase
AT1	Angiotensin II receptor type 1
B2	Bradykinin receptor type 2
β1, β2	β-Adrenoceptor 1, 2
cAMP	Cyclic adenosine monophosphate
CCK1,2R	Cholecystokinin 1 or 2 receptors
CysLT1	Cysteinyl leukotriene receptor 1
D5	Dopamine receptor type 5
DAG	Diacylglycerol
DMSO	Dimethyl sulfoxide
EPI	Experimental plasmonic index
ETA	Endothelin A
FP1	Formyl peptide receptor 1
GPCR	G protein-coupled receptors
H1, H3	Histamine receptor type 1, 3
ICG	Indocyanine green
IP_3_	Inositol-1,4,5-trisphosphate
IP_3_R	Inositol-1,4,5-trisphosphate receptors
M1,5	Muscarinic acetylcholine receptor type 1, 5
miniSOG	mini Singlet Oxygen Generator
MJH	Molecular jackhammer
P2Y1,P2Y2, P2Y6	P2 purinergic receptors type 1,2,6
PKA	Protein kinase A
PLC	Phospholipase C
PTH1	Parathyroid hormone receptor 1
SERCA	Sarcoplasmic/endoplasmic reticulum Ca^2+^-ATPase
V1(A)	Vasopressin receptor type 1(A)
